# Myeloperoxidase Deficiency Inhibits Cognitive Decline in the 5XFAD Mouse Model of Alzheimer’s Disease

**DOI:** 10.3389/fnins.2019.00990

**Published:** 2019-09-24

**Authors:** Rotem Volkman, Tali Ben-Zur, Anat Kahana, Ben Zion Garty, Daniel Offen

**Affiliations:** ^1^Department of Human Genetics and Biochemistry, Felsenstein Medical Research Center, Sackler School of Medicine, Tel Aviv University, Tel Aviv, Israel; ^2^Lempo Therapeutics Ltd., Binyamina, Israel; ^3^Sagol School of Neuroscience, Tel Aviv University, Tel Aviv, Israel

**Keywords:** myeloperoxidase, neutrophil, 5XFAD, Alzheimer’s, inflammation

## Abstract

Myeloperoxidase (MPO) is an enzyme expressed mostly by neutrophils and is a primary mediator of neutrophils oxidative stress response. While a profound body of evidence associates neutrophil-derived MPO in the pathogenesis of Alzheimer’s disease (AD), this role has not been assessed in an animal model of AD. Here, we produced hematologic chimerism in the 5XFAD mouse model of AD, with MPO deficient mice, resulting in 5XFAD with hematologic MPO deficiency (5XFAD-MPO KO). Behavioral examinations of 5XFAD-MPO KO showed significant superior performance in spatial learning and memory, associative learning, and anxiety/risk assessment behavior, as compared to 5XFAD mice transplanted with WT cells (5XFAD-WT). Hippocampal immunohistochemical and mRNA expression analyses showed significantly reduced levels of inflammatory mediators in 5XFAD-MPO KO mice with no apparent differences in the numbers of amyloid-β plaques. In addition, immunoblotting and mRNA analyses showed significantly reduced levels of APOE in 5XFAD-MPO KO. Together, these results indicate a substantial involvement of neutrophil-derived MPO in the pathology of 5XFAD model of AD and suggest MPO as a potential therapeutic target in AD.

## Introduction

Alzheimer’s disease (AD) is the most common neurodegenerative disease and the major cause of dementia in the elderly. It is characterized by a progressive cognitive decline along with multiple neuropathological features such as extracellular accumulation of amyloid-β plaques, intraneuronal accumulation of hyperphosphorylated tau protein, neuronal loss, brain atrophy, and chronic inflammation (Querfurth and Laferla, 2010).

Immune cells have long been known to be involved in AD pathogenesis and reactive glial cell were already described by Alois Alzheimer (Dahm, 2006). Astrocytes and microglial cells were extensively studied in the context of AD and are known to cluster around amyloid-β plaques. When these cells become activated, they secrete a variety of pro-inflammatory mediators, potentially causing oxidative damage to neuronal and surrounding vascular tissue, and promote chronic inflammation (Akiyama et al., 2000; Querfurth and Laferla, 2010; Czirr and Wyss-Coray, 2012). Although once regarded as less relevant to brain physiology and pathology, the role of peripheral immune cells in AD has been under growing investigation in recent years (Prinz and Priller, 2017). This role was strongly supported by genome-wide association studies (GWAS) performed in the last years identifying multiple loci within immune-related genes expressed by myeloid cells as susceptibility loci for AD (Hollingworth et al., 2011; Naj et al., 2011).

Neutrophils are the most common white blood cells in human circulation, accounting for 45–75% of the white blood cells in healthy adults. Neutrophils are phagocytic, granule-containing effector cells known as central mediators of the innate immune response. In response to infection, signals derived through tissue-resident immune cells, and the endothelium initiate neutrophil adhesion to blood vessel walls and migration toward the infected tissue. Upon activation, neutrophils are able to kill pathogens by phagocytosis, degranulation, and release of reactive oxygen species (ROS), and by formation of neutrophil extracellular traps (NETs) (Mócsai, 2013; Nauseef and Borregaard, 2014; De Oliveira et al., 2016).

Recent works have highlighted the role of neutrophils in AD pathogenesis. Compared to age matched controls, neutrophils from AD patients exhibited increased levels of pro-inflammatory cytokines in response to LPS and increased oxidative stress and lipid peroxidation levels (Vida et al., 2018). Moreover, oxidative stress levels in neutrophils from AD patients correlate with cognitive decline rate (Dong et al., 2018). Increased levels of NETs were identified in blood vessels and brain parenchyma of AD patients (Zenaro et al., 2015; Dong et al., 2018). In the 5XFAD mouse model of AD, enhanced neutrophils extravasate into the central nervous system (CNS) and migrate toward amyloid plaques (Baik et al., 2014; Zenaro et al., 2015). Moreover, brain-penetrated neutrophils were shown to promote AD-like pathology in two mice models of AD associated with releasing the pro-inflammatory cytokine IL-17, and by producing NETs. On the other hand, neutrophil depletion using anti-LY6G antibody or inhibition of neutrophil migration into the CNS resulted in improved cognition and memory, as well as reduced neuroinflammation and Amyloid-β levels (Zenaro et al., 2015). More recently, enhanced neutrophil adhesion in cortical capillaries was reported in the brain of AD model mice, resulting in reduced cerebral blood flow (Cruz Hernández et al., 2019).

The most abundant protein in neutrophils is MPO, comprising 5% of a neutrophils net weight (Winterbourn et al., 2016). MPO is a major mediator of neutrophils oxidative burst response against pathogens, and was found essential for the formation of NETs (Metzler et al., 2014). MPO catalyzes the formation of hypochlorous acid, as well as multiple other oxidative substances (Klebanoff, 2005). As such, MPO was widely suspected in promoting non-specific, oxidative damage to host tissue. Most importantly, in patients with AD, higher plasma concentrations of MPO has been reported (Tzikas et al., 2014), and MPO immunoreactive cells were identified in their brain (Gellhaar et al., 2017).

While these evidences suggest a detrimental role for neutrophil-derived MPO in AD, the effect of MPO deficiency was not investigated yet in a mouse models of AD. Here we report for the first time that induced MPO deficiency in 5XFAD mouse model of AD, markedly reduced the inflammatory response, and prevented cognitive impairment.

## Materials and Methods

### Animal Housing

C57BL/6 (Harlan), Mpo^Tm1/Lus^ [MPO KO, Jackson Laboratory (Zeng et al., 2018)] and 5XFAD [Jackson Laboratory (Oakley et al., 2006)] male mice were housed in a light-controlled environment (12-h light/dark cycle) and housed in individually ventilated cages with free access to food and water. All animal studies were authorized by the Animal Care, Use, and Review Committee of Tel Aviv University.

### Bone Marrow Cell Isolation From Donor Mice

Bone marrow was harvested from either C57BL/6 or Mpo^Tm1/Lus^ donor mice. Briefly, mice were sacrificed with CO2, and bone marrow was harvested from femurs, tibias, and humerus bones using a 27-gauge needle into sterile PBS with 0.5% Bovine serum albumin and 2mM EDTA. Cells were strained through a 70 μM filter (Falcon). Erythrocytes were lysed by incubation in ammonium chloride solution followed by centrifugation and reconstitution in sterile saline solution in a concentration of 5 × 10^6^ cells/ml.

### Recipient Mice Irradiation and Transplantation

5XFAD mice and their non-transgenic littermates were given 9 Gy (3Gy/minute) whole-body myeloablative irradiation from a gamma irradiation source (Biobeam GM). 24 h following irradiation, recipient mice were injected through the tail vain with 1 × 10^6^ bone marrow cells freshly isolated from donor mice using a 27-gauge needle. Mice were monitored and weighed every other day for the following 8 weeks.

### Behavioral Examinations

Behavioral examinations were executed starting from 7 months of age. Two weeks prior to the beginning of examinations, the mice were acclimated to a reversed light environment. Before each trial, the mice were transferred to the behavioral testing room 30 min prior to the beginning of the trial habituation. The order of the trials was counterbalanced across four treatments and genotypes. Mazes were cleaned with Virusolve+between trials. Open field, elevated plus maze, Y maze and Morris water maze tests, were recorded, monitored, and analyzed by an automated tracking system (Ethovision, Noldus).

### Open Field

Open field was performed to assess risk assessment/anxiety-related behavior. The test was conducted in a white, perspex, empty arena (50 cm × 50 cm × 40 cm). The mice were placed in the center of the arena and could explore it for 15 min. Total distance traveled, as well as the time spent in the center of the maze and the corners of the maze were measured.

### Elevated Plus Maze

Elevated plus maze was performed to assess risk assessment/anxiety-related behavior. The test was conducted in an apparatus consisting of four cross-shaped white perspex arms (35 cm × 5 cm) and was lifted 40 cm from the floor. Two arms, facing each other, were enclosed by 15 cm high walls, and while the other two arms were open. The mice were placed in the center of the maze facing a closed arm and was recorded for 7 min. The time spent in either the open or the closed arms of the maze was measured. Mice that fell off the open arms were excluded from analysis.

### Y-Maze

Two-trial Y-maze was performed to assess spatial memory. The test was conducted in a white, perspex Y-shape apparatus with arm length of 38 cm, width of 5 cm, and height of 15 cm. The test consisted of a sample trial and a test trial. In the sample trial, the mice were placed at the end of one arm of the maze facing the wall, while one arm of the maze was blocked, and could explore the two arms of the maze for 5 min. The sample trial was followed by a 5 min inter-trial interval. In the test trial, the mice were returned to the maze with all arms open for another 5 min. Novel arm entry (Number of novel arm entries/total arm entries) and novel arm exploration time (novel time exploration time/total arms exploration time) were measured for the first minute of the test trial.

### Morris Water Maze

Morris water maze was performed to assess spatial learning. The test was conducted in a pool (150 cm diameter, 30 cm deep) filled with water made opaque with skim milk. Black rectangle, triangle and circle cues were placed on the pool walls. The water temperature was 27 ± 1°C. A transparent platform (12 cm × 12 cm) was placed at the center of one quadrant of the pool, about 2 cm below the water surface. Each mouse undertook four trials per day for 5 consecutive days, starting each day from a different quadrant of the three quadrants not containing the platform. In each trial, a mouse was placed into the water and allowed to find the hidden platform for 60 s. If the mouse failed to find the platform within 60 s, it was guided to the platform and allowed to stay there for 10 s. The latency to find the hidden platform within 60 s, as well as total distance covered were recorded for each mouse. Mice that failed to find the platform were scored as have reached the platform in 60 s.

### Fear Conditioning

Fear conditioning test was performed to assess associative memory. The test was performed in a fear conditioning chamber (Ugo Basile, 17 cm × 17 cm x 25 cm). On day 1, the mice were placed in the chamber for 5 min for habituation. On day 2, the mice were placed again in the chamber for 3.5 min. After 2 min, the mice received two foot-shocks (0.8 mA, 2 s) with a 1-min interval. On day 3, the mice were placed in the chamber for 3 min. Fear conditioning was evaluated by scoring freezing behavior – the absence of all movement except for respiration.

### Tissue Collection

For immunostaining, 10 month old mice were anesthetized with ketamine/xylazine, and immediately perfused with 4% paraformaldehyde in 0.1 M phosphate buffer. The brains were fixed overnight in the same solution, and then placed in 30% sucrose solution for 48 h. The brains were kept until sectioning in PBS 0.01% Azid in 4°C.

For RNA and protein analyses, 10 month old mice were anesthetized with ketamine/xylazine and were immediately perfused with PBS. The brains were removed, and hippocampi were dissected, divided into left and right hemispheres, snap-frozen in liquid nitrogen, and stored at –80°C until use. For bone marrow RNA analysis, femur bones were collected, and bone marrow was extracted by flushing with a 27G needle into PBS. Bone marrow cells were frozen in Tri-reagent (Sigma) and stored at –80°C until use.

### Peroxidase Activity Assay

A total of 100 μl blood samples were obtained from 4 month old mice through retro-orbital withdrawal. Blood samples were analyzed for peroxidase activity in white blood cells using the ADVIA2120 automatic hematology analyzer, as described in Harris et al. (2005). The percentage of peroxidase-active cells in each sample, including neutrophils, monocytes and eosinophils were quantified.

### Immunostaining

Brains were snap frozen in 2-methylbutane cooled in liquid nitrogen and embedded in OCT before cutting. Coronal sections (10 μm) were cut using a freezing sliding microtome (Leica CM1850) and stored at –20°C until use. For S100β and IBA1 immunostaining, slides were washed two times with PBS, blocked and permeabilized with PBS 1% bovine serum albumin, 5% goat serum (Biological Industries, Israel) and 0.05% Triton-X (Sigma-Aldrich) for 1 h and incubated with S100β antibody (1:500, ab66028, Abcam) or IBA1 antibody (1:200, ab178847, Abcam) overnight in 4°C. Slides were washed 3 times with PBS and incubated with secondary goat anti mouse antibody (1/700, Alexa-Flour) for 1 h at room temperature. DNA was stained with DAPI (1:1000, Sigma-Aldrich). For Thioflavin S (ThioS) staining, slides were incubated for 8 min with 0.01% ThioS solution in 50% ethanol. Slices were then briefly incubated twice for 10 s with 80% ethanol, and washed twice with double distilled water (DDW).

### Confocal Imaging

Fluorescence images were obtained using a confocal microscope LSM710 (Carl Zeiss Micro Imaging). The confocal images were captured using a 10× and a 20× objectives (NA = 0.3, 0.8, respectively, Plan-Apochromat). Fluorescence emissions resulting from Ar 488 and 543 nm laser lines for EGFP and CY3, respectively, were detected using filter sets supplied by the manufacturer. For DAPI detection we used our mode-locked Ti: Sapphire, femtosecond pulsed, multiphoton laser (Chameleon Ultra II, Coherent, Inc.) at a wavelength of 720nm. Images were generated using the Zeiss ZEN 2011 software (Carl Zeiss, Inc.). All images were exported in TIF and their contrast and brightness were optimized using ImageJ. S100β mean intensity and ThioS plaque density were analyzed using ImageJ.

### Protein Extraction and Western Blot

Hippocampi were thawed and homogenized in lysis buffer (200 mM HEPES, 5 mM EDTA, 1% Nonidet P-40, 0.5% sodium deoxycholate, 1 mM Na2VO4, 150 mM NaCl, and 50 mM NaF) supplemented with protease inhibitor (Roche). Cells were incubated for 1 h at 4°C. Proteins were cleared by centrifugation at 14,000 × *g* for 20 min at 4°C. Protein concentrations were quantified utilizing the Pierce^TM^ BCA Protein Assay Kit (Thermo Fisher Scientific). 25 μg protein from each sample was resolved in SDS-PAGE. Nitrocellulose transferred membranes were blocked for 1 h with PBS 0.1% Tween 20 with 5% bovine serum albumin and were probed with goat anti APOE antibody (1:10,000, Chemicon) overnight in 4°C and with goat anti ACTIN antibody (1:5000, MAB1501, Milipore) for 1 h at room temperature, followed by incubation with Goat anti Mouse secondary antibody (1:5,000, Licor) for 1 h at room temperature. Visualization and analysis of band intensities were performed using the Odyssey system (Licor) and the Image Studio Lite 5.2 software. For each sample, APOE results were normalized to ACTIN.

### RNA Extraction and Real Time PCR

Hippocampal RNA was extracted using RNeasy Mini Kit (Qiagen). RNA from bone marrow samples was extracted as previously described (Rio et al., 2010) and was cleaned using RNeasy Mini Kit (Qiagen). RNA was reverse transcribed to complementary DNA (cDNA) using verso cDNA synthesis kit (Thermo Fisher Scientific). Semi-quantitative PCR was performed on the Step-One Real time PCR system using Syber-Green Master mix (Thermo Fisher Scientific) and the custom designed primers. Threshold cycle values were determined in triplicates and presented as average compared with Actin. Fold changes were calculated using the 2^–ΔΔ*CT*^ method.

### Primer Set List (All for Mouse Genes)

APOE Forward, 5′-GAGCTGATCTGTCACCTCCG-3′ and Reverse 5′-GACTTGTTTCGGAAGGAGC-3′; CCL2 Forward, 5′-GGGATCATCTTGCTGGTGAA-3′ and Reverse 5′-AGGT CCCTGTCATGCTTCTG-3′; CDH1 Forward, 5′- GGTTTTCT ACAGCATCACCG-3′ and Reverse 5′-GCTTCCCCATTTGAT GACAC-3′: CDH5 Forward, 5′-TCTTGCCAGCAAACTC TCCT-3′ and Reverse 5′-TTGGAATCAAATGCACATCG-3′; CLOUDIN5 Forward, 5′-TTTCTTCTATGCGCAGTTGG-3′ and Reverse 5′- GCAGTTTGGTGCCTACTTCA-3′: CXCL10 Forward, 5′-AACTGCATCCATATCGATGAC-3′ and Reverse 5′-GTGGCAATGATCTCAACAC-3′; IL1β Forward, 5′-GGAGA ACCAAGCAACGACAAAATA-3′ and Reverse 5′-TGGGGAA CTCTGCAGACTCAAAC-3′; GLUT1 Forward, 5′-ATGGATCC CAGCAGCAAG-3′ and Reverse 5′-CCAGTGTTATAGCCGA ACTGC-3′: MPO Forward, 5′- TGGTGGCCTGCAGAGTAT GA -3′ and Reverse 5′- GTTGAGGCCAGTGAAGAAGG -3′; OCCLUDIN Forward, 5′- AGCCTGGACATTTTGCTCAT-3′ and Reverse 5′- CATGCATCTCTCCGCCATAC-3′: S100β Forward, 5′-TTGCCCTCATTGATGTCTTCCA-3′ and Reverse 5′-TCTGCCTTGATTCTTACAGGTGAC-3′; TNFα Forward, 5′-AGGGTCTGGGCCATAGAACT-3′ and Reverse 5′-CCACCA CGCTCTTCTGTCTAC-3′; VCAM1 Forward, 5′-GGAGCC TGTCAGTTTTGAGAATG-3′ and Reverse 5′-TTGGGGA AAGAGTAGATGTCCAC-3′; ZO1 Forward, 5′- ACGACAAAA CGCTCTACAGG-3′ and Reverse 5′- GAGAATGGACTG GCTTAGCA-3′.

### Statistical Analysis

All data are expressed as the mean ± SEM. Statistical analysis was performed using GraphPad Prism 7. Behavioral data as well as bone marrow MPO mRNA levels were analyzed using unpaired student’s *t*-test. Other mRNA analyses, blotting quantification, S100β intensity quantification and ThioS plaque density were analyzed using Mann-Whitney non-parametric test. For all tests, the statistical significance threshold was set to *p* < 0.05.

## Results

### Generation of 5XFAD **Mice With** MPO **Deficiency** in the **Bone Marrow**

**FIGURE 1:**
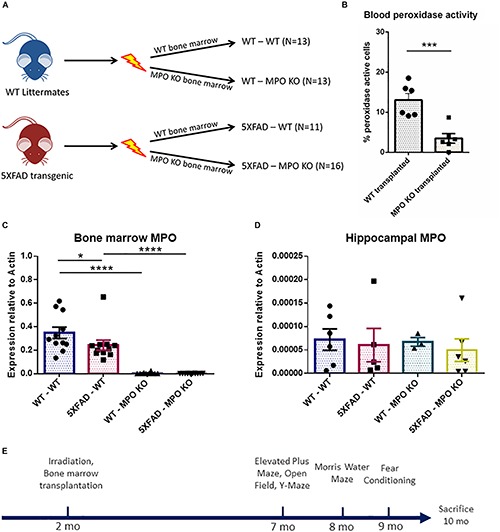
Generation of 5XFAD mice with bone marrow deficient with myeloperoxidase (MPO). Eight-week-old male 5XFAD mice and their non-transgenic littermates were lethally irradiated and transplanted with bone marrow derived from either WT C57BL/6 or MPO^tm1/lus^ mice. Numbers of mice in each group are indicated in brackets **(A)**. Peroxidase-staining cell analysis of white blood cells derived from 4 month old mice **(B)**, MPO mRNA expression analysis of bone marrow cells **(C)**, and hippocampal tissue **(D)** derived from 10-month-old mice **(B)**. Experimental design **(E)**. Data are mean ± SEM. ^∗^*P* < 0.05, ^∗∗∗^*P* < 0.001, and ^∗∗∗∗^*P* < 0.0001. Two-tailed *t*-test.

To investigate the possible role of neutrophil-derived MPO in the pathogenesis of AD, we produced hematologic MPO deficiency in adult 5XFAD mice, and their non-transgenic littermates. We subjected 2-month old mice to hematopoietic ablation by exposure to myeloablative irradiation, followed by re-population with bone marrow (BM) cells derived from either MPO-KO or WT C57BL/6 mice. This resulted in four experimental groups; (1) WT with WT BM (WT-WT), (2) WT with MPO-KO BM (WT-MPO KO), (3) 5XFAD mice with WT BM (5XAD-WT), and (4) 5XAD with MPO-KO BM (5XFAD-MPO KO) (Figure 1A). Blood peroxidase activity 2 months following transplantation revealed a substantial reduction in the percentage of cells with peroxidase activity following MPO-KO BM transplantation (Figure 1B). To further confirm the MPO reduction in mice transplanted with MPO-KO BM, we performed MPO-mRNA expression analysis. We found almost undetectable MPO-mRNA levels in BM derived from WT-MPO KO and 5XFAD-MPO KO mice, as compare to high levels in the controls indicating for efficient exchange of the hematopoietic population in the transplanted mice (Figure 1C). In order to exclude the possibility of changes in brain-derived MPO expression, we measured hippocampal MPO mRNA expression. We observed relatively low mRNA levels with no differences between our experimental groups (Figure 1D).

### MPO Deficiency Inhibit Cognitive Impairment in 5XFAD Mice

**FIGURE 2:**
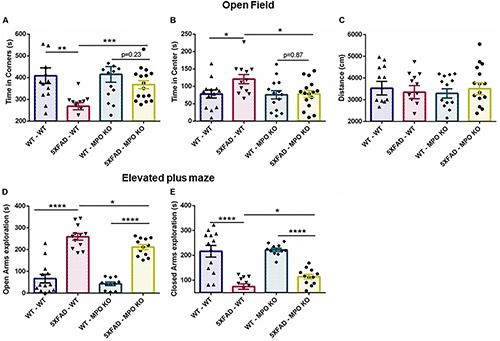
Myeloperoxidase deficiency inhibit increase in anxiety-related behavior in 5XFAD mice. Seven-month old mice were subjected to open field and elevated plus maze tests. In the open field test, time spent in the corners **(A)** and center **(B)** of the maze was measured, along with total distance traveled **(C)**. In the elevated plus maze test, time spent in either the open **(D)** or the closed **(E)** arms of the maze was measured. Data are mean ± SEM. ^∗^*P* < 0.05, ^∗∗^*P* < 0.01, ^∗∗∗^*P* < 0.001, and ^∗∗∗∗^*P* < 0.0001. Two-tailed *t*-test.

We examined the effect of MPO KO on cognitive impairment in 5XFAD mice. To this aim, we subjected the transplanted mice to a series of behavioral examinations, starting from 7 months of age (Figure 1E). In the open field test, 5XFAD mice are known to show impaired risk assessment reflected by spending more time at the center of the maze, rather than the periphery (Son et al., 2018). Indeed, 5XFAD-WT mice spent significantly less time at the corners of the maze and more time at the center of the maze, compared to WT-WT mice. In contrast, 5XFAD-MPO KO mice spent significantly less time at the center of the maze compared to 5XFAD-WT mice and exhibited a comparable behavior to as the control WT mice groups (Figures 2A,B). Notably, no difference was measure between the four experimental groups in the total distance traveled, indicating for similar motor capacity (Figure 2C).

In the elevated plus maze, WT mice tend to spend more time in the closed arms of the maze, while 5XFAD mice show tendency toward the open, exposed arms, and reflecting impaired risk/anxiety-related assessment (Schneider et al., 2014). In our experiment, both 5XFAD-WT and 5XFAD-MPO KO mice spent significantly more time in the open arms, reflecting perturbed risk assessment behavior. However, 5XFAD-MPO KO mice spent significantly less time in the open arms of the maze compared to 5XFAD-WT mice (Figures 2D,E). Together, these results indicate that MPO KO reduce the risk assessment/anxiety-related behavior in 5XFAD mice.

**FIGURE 3:**
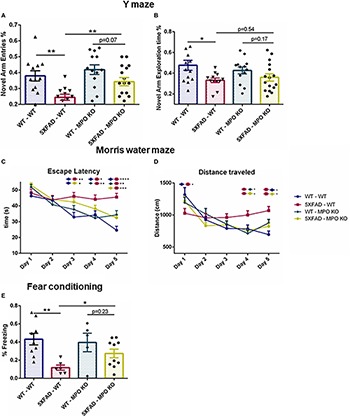
Myeloperoxidase deficiency protects against learning and memory decline in 5XFAD mice. Short-term spatial memory in 7-month old mice was assessed using a two-trial Y-maze test. Following a 5 min. sample trial in which mice were habituated to two arms of the maze, novel-arm exploration relative frequency **(A)** and time **(B)** was measured in the first 1 min. of the test trial. Spatial learning was assessed in 8-month old mice in the Morris water maze test. Mice were trained to find the hidden platform 4 times a day, for 5 days. Escape latency **(C)** and total distance covered **(D)** were measured. Associative memory was assessed in 9-month old mice by measuring freezing behavior in the fear conditioning test. Freezing behavior was measured for 3.5 min. one day following exposure to aversive foot shocks **(E)**. Data are mean ± SEM. ^∗^*P* < 0.05 and ^∗∗^*P* < 0.01. Two-tailed *t*-test.

Next, we tested whether MPO deficiency would inhibit the cognitive decline seen in 5XFAD mice. In the Y-maze test, 5XFAD-WT mice showed reduced exploration time and less entries to the novel arms as compared to WT-WT, while 5XFAD-MPO KO mice demonstrated about 50% more entries to the novel arms of the maze as compared to 5XFAD WT (Figure 3A). No significant difference in exploration time was measured between 5XFAD-WT and 5XFAD-MPO KO mice (Figure 3B). We then examined spatial learning capacity in these mice in the Morris water maze. During the 5 days of the trial, 5XFAD-WT mice showed significantly longer latency to find the platform from day 3 of the trial, reflecting impaired spatial learning (Figure 3C). However, 5XFAD-MPO KO mice showed marked better learning capacity, demonstrated by significantly less latency to find the platform at day 5, as compared to 5XFAD-WT mice (Figure 3C). The learning improvement in WT mice could also be reflected in the total distance traveled by the mice, as in early days these mice traveled a longer distance than in later days. While 5XFAD-WT mice exhibited relatively consistent traveling distance throughout the trial, 5XFAD-MPO KO mice showed shorter traveling distance as the test progressed, similar to WT groups (Figure 3D).

Finally, we assessed associative learning by using the fear conditioning test. In this test, freezing behavior was measured 1 day following conditioning with aversive foot shocks. While WT-WT and WT-MPO KO mice showed ∼40% freezing behavior, reflecting proper associative memory reconsolidation, and 5XFAD-WT mice showed significantly less freezing behavior. Notably, MPO deficient mice show higher freezing behavior in 5XFAD mice by 2.45-fold (Figure 3E). Thus, we demonstrated in five different behavioral tests, that hematologic MPO deficiency inhibit cognitive function impairment in the 5XFAD model of AD.

### MPO KO Mice Groups Show Similar Amyloid-β Plaque Load

**FIGURE 4:**
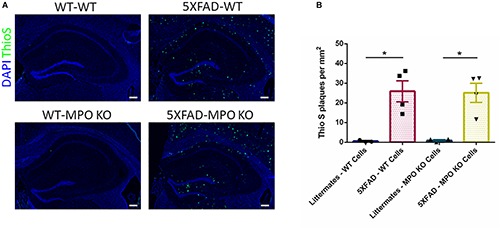
Myeloperoxidase deficiency does not affect amyloid-β plaque load in 5XFAD mice. Representative images of hippocampal slices stained with ThioS **(A)**. Quantification of ThioS plaque density **(B)**. Scale bar = 200 μm. Data are mean ± SEM. ^∗^*P* < 0.05. Two-tailed Mann-Whitney test.

Following the behavioral examinations, we sacrificed the mice and performed ThioS staining on brain sections to determine the impact of MPO KO on plaque density. While WT mice groups showed no detectable ThioS staining, both 5XFAD-WT and 5XFAD-MPO KO mice groups showed comparable high levels of amyloid-β plaques, indicating that MPO KO had no effect on the generation and accumulation of amyloid-β in the brain of 5XFAD mice (Figures 4A,B).

### 5XFAD-MPO KO Mice Show Reduced Inflammation

**FIGURE 5:**
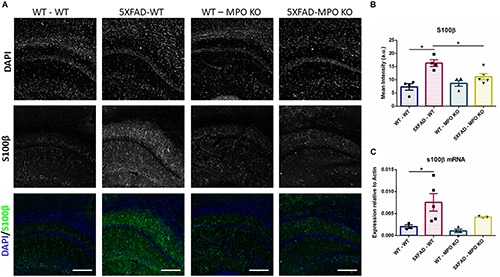
Low S100β expression in 5XFAD MPO KO mice. Representative images of hippocampal slices stained with S100β **(A)**. Quantification of S100β mean intensity **(B)**. S100β mRNA expression analysis of Hippocampal samples **(C)**. Scale bar = 200 μm. Data are mean ± SEM. ^∗^*P* < 0.05. Two-tailed Mann-Whitney test.

**FIGURE 6:**
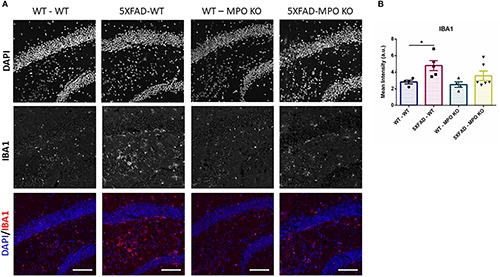
Low IBA1 expression in 5XFAD MPO KO mice. Representative images of hippocampal slices stained with IBA1 **(A)**. Quantification of S100β mean intensity **(B)**. Scale bar = 200 μm. Data are mean ± SEM. ^∗^*P* < 0.05. Two-tailed Mann-Whitney test.

To evaluate the effect of MPO deficiency on brain inflammation, we stained hippocampal sections with antibody against S100β. This protein is known to be overexpressed in activated astrocytes and has been implicated in dystrophic neurites formation in AD (Mrak et al., 1996; Sheng et al., 1996). Expectedly, 5XFAD-WT mice exhibited significantly high S100β expression in their hippocampus when compared to WT-WT mice (Figures 5A,B). However, 5XFAD-MPO KO mice exhibited low S100β levels comparable to 5XFAD-WT mice, indicating a more restricted inflammatory response in these mice. Real-time PCR (RT-PCR) analysis of hippocampal mRNA samples also showed a significant upregulation in S100β expression in 5XFAD-WT mice when compared to WT-WT mice, mirrored by a limited expression pattern in MPO deficient 5XFAD mice (Figure 5C). Next, we measured microglial activation by staining hippocampal sections with antibody against IBA1. In accordance to previous results in this model, 5XFAD-WT mice exhibited significantly elevated IBA1 expression in their hippocampus, when compared to WT-WT mice. 5XFAD-MPO KO mice have showed a 26% reduction in IBA1 staining, as compared to 5XFAD-WT mice, however this difference did not reach statistical significance (Figure 6).

**FIGURE 7:**
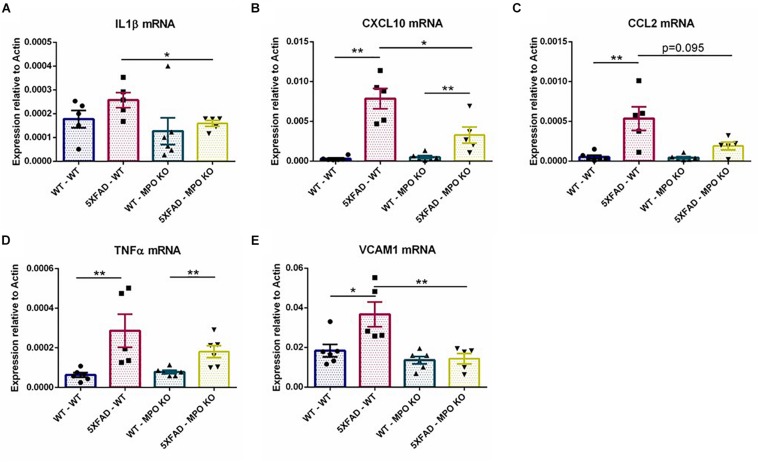
Low levels of inflammatory markers in 5XFAD MPO KO mice. mRNA expression analysis of IL1β **(A)**, CXCL10 **(B)**, CCL2 **(C)**, TNFα **(D)**, and VCAM1 **(E)** in hippocampal samples. Data are mean ± SEM. ^∗^*P* < 0.05 and ^∗∗^*P* < 0.01. Two-tailed Mann-Whitney test.

To further examine the effect of MPO deficiency on inflammation, we used RT-PCR to analyze hippocampal mRNA expression of several recognized inflammatory markers. MPO deficient 5XFAD mice showed significantly diminished expression levels of the pro-inflammatory mediators IL1β and CXCL10 (Figures 7A,B) and to some extent reduced CCL2 and TNFα expression (Figures 7C,D). Interestingly, VCAM1 levels that are enhanced in 5XFAD-WT mice were diminished in 5XFAD-MPO KO mice (Figure 7E). Given the association of VCAM1 with endothelial impairment (Kelleher and Soiza, 2013), this result may suggest a beneficial effect of MPO KO on endothelium integrity in 5XFAD mice. Further mRNA expression of transcripts coding for tight junction proteins (Cloudin5, Occludin, ZO1), adherent junction proteins (CDH1 and CDH5) and the brain endothelial marker GLUT1 did not reveal an MPO-dependent increased expression in 5XFAD mice (Supplementary Figure S1).

### MPO Deficiency Reduces APOE Expression in 5XFAD Mice

**FIGURE 8:**
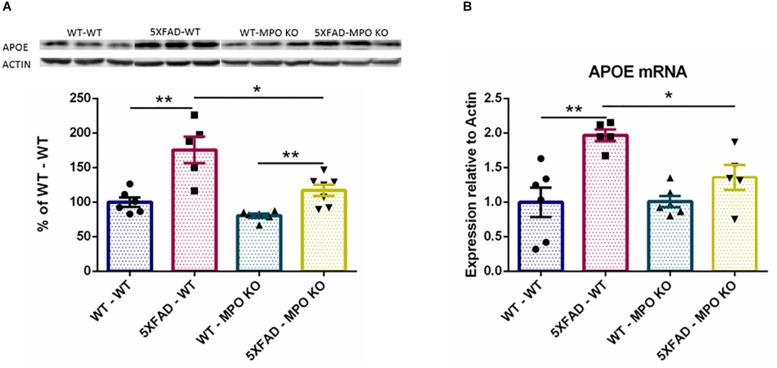
Low APOE levels in 5XFAD MPO KO mice. Western blot analysis of hippocampal cell lysates probed with anti APOE antibody **(A)**. Entire Gel image is shown in Supplementary Figure S1. mRNA expression analysis of APOE in hippocampal samples **(B)**. Data are mean ± SEM. For western blot analysis, data are represented as % of each sample to the WT-WT group average. ^∗^*P* < 0.05 and ^∗∗^*P* < 0.01. Two-tailed Mann-Whitney test.

The ε4 allele of the APOE genotype is considered as the largest genetic risk factor for sporadic late-onset AD (Corder et al., 1993; Huang and Mucke, 2012). While the exact role of APOE in AD is still under debate, APOE clearly plays a central role in AD progression and has been linked to many pathological processes relevant to AD (Liu et al., 2013), including amyloid metabolism and aggregation (Holtzman et al., 2002), and tau pathology (Shi et al., 2017). Interestingly, APOE expression was found to substantially increase in the brains of 5XFAD mice (Hong et al., 2013). Indeed, western blot analysis of hippocampal protein lysates showed a significant increase in the levels of APOE in the brains of 5XFAD-WT mice when compared to WT-WT mice, while APOE levels were significantly lower in 5XFAD-MPO KO mice (Figure 8A and Supplementary Figure S2). This result was further validated on the RNA level (Figure 8B), indicating that MPO deficiency prevented APOE elevation within the CNS.

## Discussion

In this work, we show that MPO-deficient 5XFAD exhibit superior behavioral performance compared to MPO-expressing 5XFAD mice, thus reflecting limited cognitive decline. We also show lower inflammatory response in the brains of these mice, as reflected by reduced levels of astrocytic activation, APOE expression, and the levels of several pro-inflammatory cytokines.

Central to neutrophil function, and longtime suspected in its role in neurodegeneration, MPO stands as a prominent mediator of neutrophils damage in AD. Indeed, several associations of MPO with AD have been reported in the past. It was previously shown that polymorphism in the 463G/A loci in the promoter of human MPO, linked to increased MPO expression, elevated the risk of developing late onset AD (Reynolds et al., 1999). Furthermore, an interaction was identified between MPO and the APOE ε4 allele, the most common genetic risk factor for late onset AD^41^. Following studies, however, exhibited contradicting findings regarding the association of the 463G/A polymorphism with AD (Crawford et al., 2001; Combarros et al., 2002; Leininger-Muller et al., 2003; Ji and Zhang, 2017). Green et al. (2004) have reported elevated MPO immunoreactivity and activity in the cortices of AD patients, along with increased prevalence of its oxidation products. However, MPO expression in that study was attributed to neurons. Reynolds et al. (1999) detected MPO expression in CD68 expressing cells surrounding amyloid-β plaques, therefore attributing it to microglia/macrophages. More recently, MPO expression was reported in association with amyloid-β plaques in the brains of AD patients (Gellhaar et al., 2017). Interestingly, no MPO mRNA expression was detected in those brains, indicating that the origin of MPO observed is of peripheral, myeloid nature. In the periphery, while limited MPO expression can also be detected in circulating monocytes, MPO is most abundantly expressed by neutrophils. While these observations provide compelling evidence to the involvement of MPO in AD pathogenesis, to our knowledge, our study is the first to examine the effect of MPO deficiency in an experimental model of AD.

Here we focused on peripheral MPO, predominantly derived from neutrophils by utilizing hematopoietic depletion and repopulation methods, to establish MPO deficiency in peripheral myeloid cells, without affecting CNS-derived MPO. Yet, it should be mentioned that following irradiations some HSC-derived myeloid cells might repopulate the microglial niche (Hess et al., 2004). Thus, although we could not detect any difference in CNS derived microglial MPO expression between 5XFAD-WT and 5XFAD-MPO KO mice, we cannot totally exclude such effect.

Oxidative stress and vascular damage are considered key events in the pathogenesis of AD. These can potentially lead to blood brain barriers dysfunction, decreased cerebral blood flow, and substantial oxidative and inflammatory damage within the CNS (Zlokovic, 2011). Recent implications for neutrophil function in neurodegeneration include hyper-activation and senescence (Vida et al., 2018), enhanced adhesion in cortical capillaries (Cruz Hernández et al., 2019), extravasation and degranulation in the brain parenchyma and the formation of NETs, and leading to profound inflammatory response (Zenaro et al., 2015). Our data also demonstrate that hematological MPO deficiency limit the inflammatory response. In addition, the profound protection against cognitive decline following MPO depletion may account for the reduced oxidative damage derived from neutrophils, which consider a substantial part of the pathology in AD. In this context, our results are in line with recent publications by Zenaro et al. (2015) and Cruz Hernández et al. (2019) exhibiting functional protection in mouse models of AD following neutrophil depletion.

Myeloperoxidase function have been implicated in endothelial dysfunction and vascular damage in the context of cardiovascular (Baldus et al., 2004; Vita et al., 2004; Teng et al., 2017) and kidney diseases (Lehners et al., 2014; Kisic et al., 2016; Zeng et al., 2018). Positively charged MPO secreted by activated neutrophils accumulates along the endothelium and subendothelial space, through its interaction with anionic glycocalyx (Baldus et al., 2001). While oxidants derived from bound MPO were shown to reduce glycocalyx thickness and damage to the endothelium (Üllen et al., 2013; Manchanda et al., 2018), MPO was also shown to promote neutrophil recruitment and transmigration (Klinke et al., 2011). This may result in amplified MPO response, and *in situ* accumulation of neutrophils. Interestingly, we found a decrease in VCAM1 expression in hippocampal samples of MPO deficient 5XFAD mice, suggesting a decrease in inflammation in the endothelium. However, the effect of MPO deficiency on recruitment of neutrophils in microvascular endothelium and its effects on capillary stalls and cerebral blood flow, and neutrophil migration, are yet to be determined.

APOE expression is elevated in the brains of 5XFAD mice (Hong et al., 2013), and in the brains of AD patients, regardless of the allelic variance (Gottschalk et al., 2016). Brain APOE expression is to large extent attributed to astrocytic cells, and was associated with astrocytic Aβ phagocytosis and degradation (Koistinaho et al., 2004). Recent reports have also highlighted the upregulation of APOE in microglial cells involved in neurodegeneration (Keren-Shaul et al., 2017; Krasemann et al., 2017). In this context, APOE was shown to suppress homeostatic microglial phenotype, resulting in enhanced inflammatory response (Krasemann et al., 2017). In this study, while we could not detect a significant difference in microglial activation, MPO deficiency have reduced astrocytic hyperactivation, and suggesting that APOE reduction was due to limited atrocytic pro-inflammatory response.

In humans, MPO deficiency is a caused by mutations in the MPO gene on chromosome 17, with estimated frequency of 1 per 2,000 to 1 per 4,000 individuals in the United States and Europe (Ren and Fedoriw, 2012). While MPO is central to neutrophil function and is essential for the formation of NETs (Metzler et al., 2014), 95% of the individuals with MPO deficiency were reported to be asymptomatic (Ren and Fedoriw, 2012), and indicating that MPO inhibition may be, to a wide extent, tolerable for proper immune function. Recently, a small molecule inhibitor of MPO (AZD3241) was shown to be safe and tolerable in healthy adults and patients with Parkinson’s disease (Posener et al., 2014) and is currently under clinical investigation for the treatment of Multiple System Atrophy. These lines of evidence attribute MPO as a suitable target for intervention.

## Conclusion

In conclusion, our results indicate that neutrophil-derived MPO plays a central role in the AD-like pathogenesis in 5XFAD mice. These results propose MPO inhibition as a novel therapeutic target for the treatment of AD.

## Data Availability

All datasets generated for this study are included in the manuscript and/or the Supplementary Files.

## Ethics Statement

The animal study was reviewed and approved by the Animal Care, Use, and Review Committee of Tel Aviv University.

## Author Contributions

RV, AK, TB-Z, BG, and DO designed the experiments and edited the manuscript. RV performed the experiments. RV and DO wrote the manuscript.

## Conflict of Interest Statement

This study was partially funded by the Lempo Therapeutics Ltd. (Binyamina, Israel). AK and BG were employed by the company Lempo Therapeutics Ltd. and are shareholders of the Lempo Therapeutics Ltd. The remaining authors declare that the research was conducted in the absence of any commercial or financial relationships that could be construed as a potential conflict of interest.

## Supplementary Material

FIGURE S1 mRNA expression analysis of Occludin **(A)**, Cloudin5 **(B)**, CDH1 **(C)**, CDH5 **(D)**, GLUT1 **(E)**, and ZO1 **(F)** in hippocampal samples. Data are mean ± SEM. ^∗^*P* < 0.05, ^∗∗^*P* < 0.01, ^∗∗∗^*P* < 0.001, and ^∗∗∗∗^*P* < 0.0001. Two-tailed Mann-Whitney test.

FIGURE S2 Full gel image of APOE western blot analysis shown in Figure 7A.
