# Supplementary figures and images for: Myeloperoxidase Deficiency Inhibits Cognitive Decline in the 5XFAD Mouse Model of Alzheimer’s Disease
Source: Front Neurosci. 2019 Sep 24;13:990. doi: 10.3389/fnins.2019.00990 (PMC6769081; doi:10.3389/fnins.2019.00990)

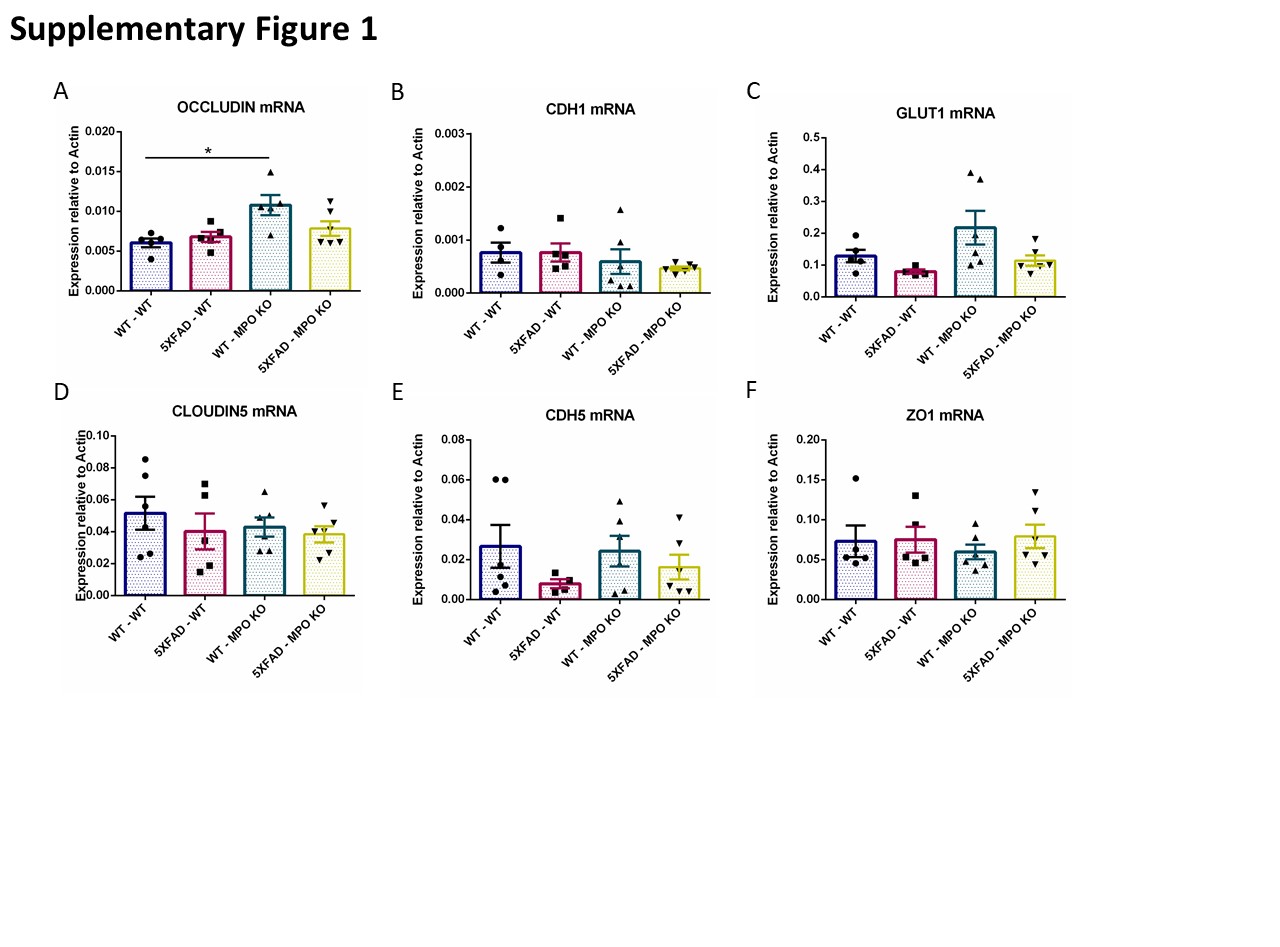

Supplement: FIGURE S1 — mRNA expression analysis of Occludin (A), Cloudin5 (B), CDH1 (C), CDH5 (D), GLUT1 (E), and ZO1 (F) in hippocampal samples. Data are mean ± SEM. ∗P < 0.05, ∗∗P < 0.01, ∗∗∗P < 0.001, and ∗∗∗∗P < 0.0001. Two-tailed Mann-Whitney test. [file Image_1.JPEG]

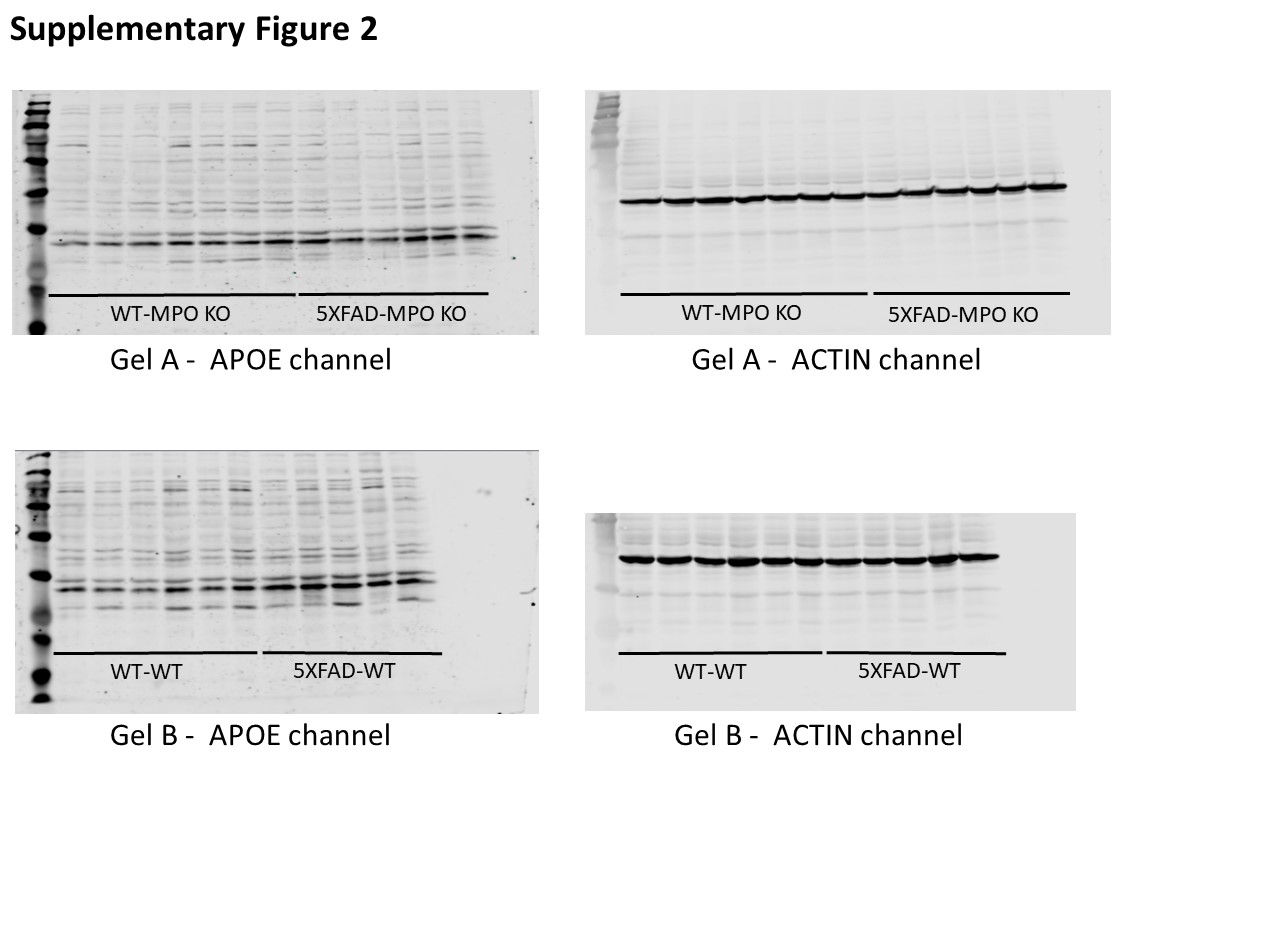

Supplement: FIGURE S2 — Full gel image of APOE western blot analysis shown in Figure 7A. [file Image_2.JPEG]
